# Improved efficiency using sequential automated immunoassays for syphilis screening in blood donors

**DOI:** 10.1128/jcm.00476-24

**Published:** 2024-07-15

**Authors:** Anthea Cheng, Anindita Das, Claire E. Styles, Zin Naing, William D. Rawlinson, Iain B. Gosbell

**Affiliations:** 1 Pathology and Clinical Governance, Australian Red Cross Lifeblood, West Melbourne, Victoria, Australia; 2 Clinical Microbiology, ACT Pathology, Garran, Australian Capital Territory, Australia; 3 Faculty of Health, University of Canberra, Bruce, Australian Capital Territory, Australia; 4 Serology and Virology Division (SAViD), NSW Health Pathology, Randwick, New South Wales, Australia; 5 Virology Research Laboratory, Prince of Wales Hospital, Randwick, New South Wales, Australia; 6 School of Medical Sciences, University of New South Wales, Sydney, New South Wales, Australia; 7 School of Biotechnology and Biomolecular Sciences, University of New South Wales, Sydney, New South Wales, Australia; 8 School of Medicine, Western Sydney University, Penrith, New South Wales, Australia; Medical College of Wisconsin, Milwaukee, Wisconsin, USA

**Keywords:** syphilis, sequential immunoassays, blood donor screening

## Abstract

Using sequential immunoassays for the screening of blood donors is well described for viral serology testing but not for the screening of syphilis. In this study, we report the evaluation results and 2-year sequential testing data using two highly sensitive automated serology assays, the Alinity s Syphilis chemiluminescent immunoassay for screening, with all repeatedly reactive samples then tested on the Elecsys Syphilis electrochemiluminescence immunoassay. We screened 1,767,782 blood donor samples between 7 July 2021 and 6 July 2023 and found the Alinity false-positive rate to be low at 0.08% (1,456/1,767,782). The common false-positive rate between the two assays was also low (3.83%, 58/1,514). Concordantly reactive samples were further tested using a *Treponema pallidum* particle agglutination test, a rapid plasma reagin test, and a fluorescent treponemal antibody absorption test. There were 262/1,376 concordantly reactive Alinity and Elecsys blood donor samples with reactivity on one or more of the confirmatory tests. A total of 26/1,376 donors had a current syphilis infection, 152/1,376 reported a past history of syphilis and had been treated, and 84/1,376 did not report a past history of syphilis. We suggest that future studies could explore the use of sequential immunoassays to aid in the serodiagnosis for syphilis.

**IMPORTANCE:**

The serodiagnosis for syphilis usually follows two methodologies—a “traditional” algorithm using a non-treponemal test followed by confirmation using a treponemal test, or a “reverse” algorithm using a treponemal test followed by a non-treponemal test. There are limited reports in the literature of using a modified reverse algorithm (treponemal test followed by a second treponemal test), and to the best of knowledge, there are currently no published articles using two highly sensitive automated immunoassays to aid the serodiagnosis of syphilis. In addition, the *Treponema pallidum* particle agglutination (TPPA) assay is commonly used as a confirmatory test for the diagnosis of syphilis. With the withdrawal of the TPPA assay from Australia and presumably from the global market also, alternative testing algorithms are now required. This study provides proof of concept for using sequential immunoassays in the diagnosis of syphilis.

## INTRODUCTION

Syphilis was the first infection shown to be transmitted by blood transfusion, probably because the Wasserman reaction was the first available test for an agent transmitted by transfusion. Notably, most transfusion transmissions occurred in the period between the two world wars. Blood banks introduced the Wasserman test in the 1940s (1) and then other serological tests for syphilis as technology developed. The number of cases of syphilis transmitted by transfusion fell after the Second World War, with last reported cases in high-income countries occurring in the United States in 1966 and the Netherlands in 1977 (2, 3). This reduction is multifactorial and include improved donor selection processes, introducing serological testing of donors and the transfusion of refrigerated blood components rather than fresh blood (1). Some have argued that syphilis testing of blood donors could now be stopped in the face of this evidence (4, 5). Additionally, it has been suggested that serologic testing for syphilis is insensitive for the prevention of transfusion transmission as individuals are spirochetemic and potentially infectious before they develop antibodies (4). However, there is little appetite among legislative and regulatory bodies to stop syphilis screening in the midst of a worldwide epidemic of syphilis with active infections being seen in both new and returning blood donors. Internationally, blood services have retained a syphilis screening strategy using the most cost-effective and clinically effective testing available.

There have been significant technological advances in the testing for syphilis, beginning with direct demonstration of spirochetes (e.g., darkfield microscopy) and development of blood tests: reaginic or non-treponemal tests [e.g., Venereal Disease Research Laboratory (VDRL) test and rapid plasma reagin (RPR)], then treponemal tests [e.g., *Treponema pallidum* immobilization test, fluorescent treponemal antibody absorption (FTA-ABS), *T. pallidum* hemagglutination assay (TPHA), *T. pallidum* particle agglutination (TPPA), and various automatable immunoassay formats], and most recently nucleic acid tests for detection in lesions and chancres.

Syphilis serology using a combination of treponemal and non-treponemal assays is the mainstay of laboratory diagnostics. Treponemal tests detect treponema-specific antibodies and are sensitive in detecting very early syphilis. Individuals may be test-reactive for life; therefore, treponemal tests are not useful in differentiating between early and latent infections. Non-treponemal tests detect antibodies to lipoidal material produced due to treponemal infection and are more reliable indicators of untreated infection but are non-specific (6). The two types of tests can be combined to form the “traditional” and “reverse” syphilis testing algorithms.

In the traditional algorithm, a non-treponemal test is used as the screening assay with all reactive samples confirmed using a treponemal test. As non-treponemal assays are less sensitive, there is a risk that both early and latent infections may go undetected (7). While this algorithm is a cost-effective approach to diagnosing syphilis, non-treponemal tests are also less specific and are performed manually, and the results are subject to interpretation.

The reverse algorithm uses a treponemal assay first, with all reactive samples then confirmed using a non-treponemal test. Discordant results are usually resolved with a second treponemal test (8). Methods such as TPHA and treponemal chemiluminescent microparticle immunoassays (CMIAs) can be automated on high-throughput analyzers making them suitable for high-volume laboratories; however, it has been reported this algorithm can be prone to higher false-positive rates in low-prevalence populations (9). A modification of the reverse algorithm using two treponemal tests sequentially has also been described (10, 11).

For over two decades, Australian Red Cross Lifeblood (Lifeblood) used TPHA (Beckman Coulter PK TP System) for donor screening followed by an RPR test. Positive samples were then sent to local reference laboratories for confirmatory testing where a combination of treponemal and non-treponemal tests was used. The plasma from donations, which are determined to be TPHA-false positive or from donors with adequately treated past syphilis infection, is accepted for fractionation purposes only.

Following a change in the Australian regulation for *in vitro* diagnostics medical devices in July 2015, Lifeblood was unable to source an appropriately registered RPR test or equivalent test suitable for testing blood donor samples. As a consequence, from April 2016, TPHA-reactive samples were sent to the reference laboratory for confirmatory testing resulting in increased costs, a longer time to obtain results, and plasma wastage as false-positive results could not be determined internally.

The use of sequential screening immunoassays 1 and 2 (IA1 and IA2) to clarify the antibody status of blood donors is successful in viral antibody testing algorithms but has not been applied to syphilis screening. As blood donors have to pass the donor questionnaire which asks about infectious risks, the pre-test probability of the various infections screened for is low, and thus there are a considerable proportion of false-positive tests in blood donors. Using sequential immunoassays that have sufficient differences in detection systems, assay format, and antigens is a cost-effective method to differentiate false-positive from true positive results without impacting sensitivity (12). As described by Seed et al., the effectiveness of this strategy requires careful selection of the assay combination to minimize the false-positive overlap. The selected IA1 should be highly specific to ensure that false-positive reactions are minimized at the primary screening assay level, and the selected IA2 should have comparable sensitivity to the IA1, to reduce the chance of false-negative results. An efficient combination of assays will result in enhanced specificity by reducing the number of false-positive results requiring confirmatory tests. As the majority of assay manufacturers do not disclose the specific details of the antigens or the antigenic domain used in their assay, evaluation of candidate assays with pedigreed samples is required before implementation, with ongoing monitoring of results following implementation.

Adopting this principle, Lifeblood introduced a CMIA assay (ARCHITECT Syphilis TP) in December 2018 as the sequential treponemal test (IA2) to the TPHA (13). Two years later in December 2020, the TPHA screening assay was replaced with the Alinity s Syphilis CMIA (IA1). The use of the ARCHITECT Syphilis TP as the IA2 ceased at this point as it was expected that using two assays from the same manufacturer with similar if not identical recombinant *T. pallidum* antigens for antibody capture and the same CMIA technology would result in highly concordant results with overlap in non-specific reactivity. At the time, there were insufficient resources to source and implement an alternate IA2, and therefore, all Alinity reactive samples were referred to a reference laboratory, New South Wales (NSW) Health Pathology, for confirmatory testing.

We describe two sets of results: (i) evaluation of the cobas Elecsys Syphilis electrochemiluminescence immunoassay (ECLIA) as a suitable IA2 to the Alinity s Syphilis CMIA and (ii) 2 years of blood donor syphilis screening results using this assay combination.

## MATERIALS AND METHODS

From December 2020, samples were tested using the Alinity s Syphilis assay on the Alinity s System (Abbott GmbH & Co. KG, Wiesbaden, Germany) at one of the four Lifeblood processing centers located at Sydney, Melbourne, Brisbane, and Perth. Testing was performed according to the manufacturer’s instructions. Samples with an initial sample to cut-off (S/CO) ratio of <1.00 were considered to be negative for antibodies to *T. pallidum*, while samples with an initial S/CO of ≥1.00 were re-tested in duplicate to comply with manufacturer and regulatory requirements. If both results had a S/CO of <1.00, the sample was considered negative. If one or both results had a S/CO of ≥1.00, the sample was considered repeatedly reactive.

Alinity repeatedly reactive samples were sent to NSW Health Pathology located at Prince of Wales Hospital in Randwick for diagnostic testing. Samples were tested using the ARCHITECT Syphilis TP assay on the ARCHITECT iSystem (Abbott GmbH & Co. KG, Wiesbaden, Germany) and were also tested using Serodia-TPPA (Fujirebio Inc., Tokyo, Japan), Macro-Vue RPR (Becton, Dickinson and Company, Maryland, USA), and IFA FTA-ABS Test System (Zeus Scientific Inc., New Jersey, USA). All testing and result interpretation were performed according to the manufacturers’ instructions.

As the Alinity and ARCHITECT assays are produced from the same manufacturer and both use CMIA as the detection system, a high overlap of non-specific reactivity using these two assays in combination was expected. Therefore, an evaluation for another sequential syphilis assay was performed.

A total of 129 samples concordantly reactive on the Alinity and ARCHITECT test combination in April and May 2021 were de-identified and tested using the Elecys Syphilis assay on the cobas e 411 immunoassay analyzer (Roche Diagnostics GmbH, Mannheim, Germany). Samples with an initial cut-off index (COI) of ≥1.00 were considered to be reactive, and those with a COI of <1.00 were considered to be non-reactive.

The Elecsys results were then assessed against pre-determined syphilis outcomes derived from the results of the three confirmatory tests (TPPA, RPR titer, and FTA-ABS), shown in Table 1.

**TABLE 1:** Pre-determined syphilis outcomes based on confirmatory test results^*a*^

Screen	Confirmatory tests				Number	Pre-determined syphilis outcome
Alinity	ARCHITECT	TPPA	RPR	FTA-ABS		
RR	R	NR	NR	NR	100	Syphilis not confirmed^*b*^
RR	R	R	R	R	10	Syphilis confirmed^*c*^
RR	R	R	NR	R	10	Syphilis confirmed^*c*^
RR	R	NR	NR	R/Wk	6	Syphilis likely^*d*^
RR	R	R	NR	NR	3	Syphilis likely^*e*^

^*a*^ TPPA, *T. pallidum* particle agglutination; RPR, rapid plasma reagin; FTA-ABS, fluorescent treponemal antibody absorption; RR, repeatedly reactive; R, reactive; NR, non-reactive; Wk, weakly reactive.

^*b*^ Alinity and ARCHITECT false-positive likely.

^*c*^ Current or past infection suspected.

^*d*^ This profile may also be found in Lyme disease, herpes simplex virus infection, seroreversion, and other endemic treponematoses (e.g., Yaws and Pinta).

^*e*^ This may indicate seroreversion.

Following the completion of the evaluation, a new syphilis testing algorithm was established. From 12 July 2021, samples repeatedly reactive on the Alinity (IA1) at Lifeblood were referred to NSW Health Pathology for the sequential test using the Elecsys assay (IA2). Only samples that were reactive on both the IA1 and IA2 were further tested using TPPA, RPR, and FTA-ABS. Samples reactive on the Alinity (IA1) and non-reactive on the Elecys (IA2) were considered to be Alinity false-positive. Donors with any other pattern of results were contacted by Lifeblood to obtain a history and to elicit any risk factors. Donors who did not report a history of past treated syphilis infection were either referred to their general practitioner for follow-up or asked to return to Lifeblood for repeat testing. When available, repeat syphilis serology results were included for outcome assessment.

First-time Alinity false-positive donors are not contacted until they return two false-positive results within 12 months. When this occurs, the donor is informed of their false-positive result and asked to donate for apheresis plasma for fractionation only, where they will no longer be tested for syphilis.

The efficiency of IA1/IA2 combinations can be assessed by determining the false-positive overlap between the two assays. False-positive overlaps between the IA1 and candidate IA2s have been shown to vary considerably for viral antibody assays and where possible, which should have a maximum false-positive population overlap of approximately 5% (12). The percentage of false-positive overlap between the IA1 and IA2 was estimated using the following general formula:


$$\frac{Total\;number\;of\;samples\;reactive\;on\;both\;primary\;and\;sequential\;assay\;but\;not\;confirmed\;as\;positive}{Total\;number\;of\;samples\;reactive\;on\;primary\;assay\;but\;not\;confirmed\;as\;positive}\;\times \;100.$$

## RESULTS

### Evaluation of Elecsys syphilis ECLIA as the sequential assay

A total of 129 concordantly reactive Alinity and ARCHITECT samples were tested on the Elecsys with a syphilis outcome derived on the results of the three confirmatory tests (TPPA, RPR titer, and FTA-ABS; Table 2). Syphilis was not confirmed in 100 samples, and the majority (97.0%) were non-reactive on the Elecsys. This pattern of results is suggestive of Alinity false-positive although early infection cannot be excluded. Of the 29 samples where syphilis was confirmed or likely with minimum one reactive confirmatory test, 21 (72.4%) were Elecsys reactive. For the eight samples that were Elecsys non-reactive, six were solely reactive or weakly reactive on FTA-ABS assay, and two were solely reactive on TPPA.

**TABLE 2:** Test results from Elecsys syphilis ECLIA and the derived outcome based on the confirmatory test results^*a*^

Pre-determined syphilis outcome	IA1	IA2	Confirmatory tests			Derived outcome using IA2 and confirmatory test results	Number (percentage)
	Alinity	Elecsys	TPPA	RPR	FTA-ABS		
Not confirmed	RR	NR	NR	NR	NR	Alinity false-positive	97 (75.2)
Likely	RR	NR	NR	NR	R/Wk	Elecsys false-negative	6 (4.7)
Likely	RR	NR	R^*b*^	NR	NR	Elecsys false-negative	2 (1.6)
Not confirmed	RR	R	NR	NR	NR	Alinity and Elecsys false-positive	3 (2.3)
Confirmed	RR	R	R	R	R	Confirmed	10 (7.8)
Confirmed	RR	R	R	NR	R	Confirmed	10 (7.8)
Likely	RR	R	R^*c*^	NR	NR	Confirmed	1 (0.8)

^*a*^ IA1, immunoassay 1; IA2, immunoassay 2; TPPA, *T. pallidum* particle agglutination; RPR, rapid plasma reagin; FTA-ABS, fluorescent treponemal antibody absorption; RR, repeatedly reactive; NR, non-reactive; R, reactive; Wk, weakly reactive.

^*b*^ TPPA titer of 160 and 80.

^*c*^ TPPA titer of 320.

### Two-year results using Elecsys syphilis ECLIA as the sequential assay

Between 7 July 2021 and 6 July 2023, 1,767,782 samples were screened with the Alinity syphilis assay. A total of 1,850 samples (0.10%) were found to be repeatedly reactive, and 1,456 of these tested non-reactive on the Elecsys (IA2). The presumed Alinity false-positive rate was determined to be 0.08% (1,456/1,767,782).

The 1,850 samples were collected from 1,376 donors. The syphilis results for these donors are shown in Table 3. For donors with multiple samples tested during the period, only the most recent or significant result is shown.

**TABLE 3:** Syphilis results from 1,376 donors tested between 7 July 2021 and 6 July 2023 and the assigned outcomes based on confirmatory test results and donor counseling^*a*^

IA1	IA2	Confirmatory tests			Assigned outcome	Number (percentage)
Alinity	Elecsys^*b*^	TPPA	RPR	FTA-ABS		
RR	NR	NT	NT	NT	Alinity false-positive	1,061 (77.1)
RR	R	NR	NR	NR	Alinity and Elecsys false-positive	53^*c*^ (3.8)
RR	R	R	R	R	Current infection	26^*d*^ (1.9)
RR	R	R	NR	R/Wk	Past treated	123 (8.9)
RR	R	R	R	R/Wk	Past treated	23 (1.7)
RR	R	R	NR	NR	Past treated	5 (0.4)
RR	R	NR	NR	NR	Past treated	1^*e*^ (0.1)
RR	R	R	NR	R/Wk	Unknown, referred	61 (4.4)
RR	R	R	R	R/Wk	Unknown, referred	20 (1.5)
RR	R	R	NR	NR	Unknown, referred	2 (0.1)
RR	R	NR	NR	R	Unknown, referred	1 (0.1)

^*a*^ IA1, immunoassay 1; IA2, immunoassay 2; TPPA, *T. pallidum* particle agglutination; RPR, rapid plasma reagin; FTA-ABS, fluorescent treponemal antibody absorption; RR, repeatedly reactive; NR, non-reactive; NT, not tested; R, reactive; Wk, weakly reactive.

^*b*^ Samples with an initial COI of ≥1.30 were considered to be reactive. Only samples with an initial COI of ≥1.00 to 1.30 were repeated in duplicate. If both repeated results had a COI of <1.00, the sample was considered non-reactive. If one or both repeated results had a COI of ≥1.00, the sample was considered reactive.

^*c*^ Seventeen donors (1.2%) have not returned for re-testing. Classified as Alinity and Elecsys false-positive but early infection cannot be discounted.

^*d*^ One donor tested RPR non-reactive.

^*e*^ Donor later disclosed they had tested positive for syphilis in the past.

#### Common false-positive overlap between the Alinity and Elecsys syphilis assays

A total of 1,514 samples not confirmed for syphilis were included in the analysis, with 58 samples reactive on both assays. The false-positive overlap was calculated as 3.83%.

## DISCUSSION

The current algorithm of syphilis testing using sequential immunoassays, screening with Alinity (IA1) followed by Elecsys (IA2), improved specificity without an impact on sensitivity. In our study using blood donors, we calculated the false-positive rate for the Alinity as 0.08%. This rate is comparable to our earlier assay combination using TPHA and ARCHITECT (0.14%, data not shown). During evaluation, there was a signal that a small proportion of samples (8/129) that were IA1 repeatedly reactive/IA2 non-reactive tested reactive with one of the three confirmatory tests (TPPA reactive *n* = 2, FTA-ABS reactive/weakly reactive *n* = 6). None of these were RPR reactive and are unlikely to represent infectious syphilis. These samples may represent false-negative IA2, false-positive TPPA, or FTA-ABS results, or the early stages of infection where some markers are below the limit of assay detection. As the samples were de-identified for the evaluation, we were unable to determine if the donor returned for subsequent donations and re-screened for syphilis. However, in our experience with following up blood donors for risk factor assessment and further testing, it has been observed that these patterns of results resolve over time and represent IA1 false reactivity.

The weaknesses of the study are (i) the initial 129 concordantly reactive Alinity and ARCHITECT samples that were not randomly assigned and (ii) the absence of testing TPPA/RPR/FTA-ABS on the samples testing IA1 repeatedly reactive but IA2 non-reactive during the 2-year follow-up. These were due to (i) the emphasis of the initial study that was mainly on ARCHITECT reactive samples that were not confirmed on syphilis confirmatory testing (*n* = 100) and (ii) insufficient resources to test an additional 1,456 samples with all three confirmatory tests during the 2-year follow-up study. Classifying samples that are syphilis screening reactive but non-reactive on a second test as false-positive is not uncommon in blood services (14).

There are no reports in the literature describing sequential automated immunoassays for the serodiagnosis of syphilis. One study does describe the use of the modified reverse algorithm where a treponemal test (TPPA) was used for screening with all reactive samples then tested on another treponemal assay (automated chemiluminescence immunoassay) (15). By using immunoassays from different vendors, the detection system, assay format, and antigenic domains will be likely different, noting that the precise nature of the antigens is not always available due to commercial-in-confidence considerations. Lifeblood already uses the sequential immunoassay format for the viral serological testing for human immunodeficiency virus, hepatitis C virus, and human T-lymphotropic virus. The sequential immunoassay is selected after evaluation, using the principles in Seed et al. (12), which recommends selecting two immunoassays that have a maximum common false-positive overlap of 5%. The false-positive overlap between Alinity and Elecsys was 3.83%, which suggests that this combination is suitable for sequential immunoassays.

Our study provides proof of concept for using sequential immunoassays in the diagnosis of syphilis. For blood services, this allows for most of the testing to be done on automated platforms, resulting in workflow efficiencies and assisting in donor counseling as the true positive and false-positive results are rapidly resolved. There were a small number of samples in the evaluation that tested IA1 repeatedly reactive, IA2 non-reactive, and TPPA reactive but RPR non-reactive. These are unlikely to represent infectious syphilis and are of low consequence for blood component safety; therefore, we progressed to full implementation of the sequential immunoassay algorithm. There have been no confirmed cases of blood transfusion transmission of syphilis since 1977, likely due to modern blood donation processing not facilitating survival of *T. pallidum* among other reasons, and several have also argued that syphilis testing could be dropped altogether (4, 5). For the viral agents where Lifeblood uses sequential immunoassays, there have been no lookback investigations triggered by any IA1 repeatedly reactive/IA2 non-reactive donors in over two decades of running these algorithms (data not shown).

For diagnostic laboratories, where the purpose is the diagnosis of syphilis rather than blood product safety, a higher level of specificity, clinical correlation, risk assessment, and follow-up testing is required. So, in the diagnostic setting, the concept of sequential immunoassays lends itself to automation and probable efficient workflows especially through increasing specificity without impacting sensitivity. We suggest that the concept requires further exploration, particularly around the true result for samples testing IA1 reactive/IA2 non-reactive. Noting that all syphilis tests have suboptimal sensitivity and specificity varying with the stage of infection, we suggest future research to establish the proportion of IA1 reactive/IA2 non-reactive that are reactive on other syphilis tests (e.g., TPPA/TPHA, RPR/VDRL, and FTA-ABS) to determine the overall sensitivity and specificity of the different combinations and also the costs and turnaround times to produce the best algorithm for the serodiagnosis of syphilis.

## Data Availability

The data presented in this study are available in Datasets S1; S2.

## SUPPLEMENTAL MATERIAL

Dataset S1 jcm.00476-24-s0001.xlsx Elecsys evaluation.

Dataset S2 jcm.00476-24-s0002.xlsx Two-year results.

ASM does not own the copyrights to Supplemental Material that may be linked to, or accessed through, an article. The authors have granted ASM a non-exclusive, world-wide license to publish the Supplemental Material files. Please contact the corresponding author directly for reuse.

## References

[1] Desforges JF, Athari F, Cooper ES, Johnson CS, Lemon SM, Lindsay KL, McCullough J, Mcintosh K, Ross RK, Whitsett C, et al.. 1995. Infectious disease testing for blood transfusions: NIH consensus development panel on infectious disease testing for blood transfusions. JAMA 274:1374–1379. doi:10.1001/JAMA.1995.03530170054032 7563563

[2] Chambers RW, Foley HT, Schmidt PJ. 1969. Transmission of syphilis by fresh blood components. Transfusion 9:32–34. doi:10.1111/j.1537-2995.1969.tb04909.x 5762537

[3] Risseeuw-Appel IM, Kothe FC. 1983. Transfusion syphilis: a case report. Sex Transm Dis 10:200–201. doi:10.1097/00007435-198311000-00009 6665664

[4] Katz LM. 2009. A test that won't die: the serologic test for syphilis. Transfusion 49:617–619. doi:10.1111/j.1537-2995.2009.02119.x 19335375

[5] Jayawardena T, Hoad V, Styles C, Seed C, Bentley P, Clifford V, Lacey S, Gastrell T. 2019. Modelling the risk of transfusion-transmitted syphilis: a reconsideration of blood donation testing strategies. Vox Sang 114:107–116. doi:10.1111/vox.12741 30565234

[6] Pillay A. 2018. Centers for disease control and prevention syphilis summit-diagnostics and laboratory issues. Sex Transm Dis 45:S13–S16. doi:10.1097/OLQ.0000000000000843 30102681 PMC11462382

[7] Satyaputra F, Hendry S, Braddick M, Sivabalan P, Norton R. 2021. The laboratory diagnosis of syphilis. J Clin Microbiol 59:e0010021. doi:10.1128/JCM.00100-21 33980644 PMC8451404

[8] Ortiz DA, Shukla MR, Loeffelholz MJ. 2020. The traditional or reverse algorithm for diagnosis of syphilis: pros and cons. Clin Infect Dis 71:S43–S51. doi:10.1093/cid/ciaa307 32578864 PMC7312234

[9] Binnicker MJ, Jespersen DJ, Rollins LO. 2012. Direct comparison of the traditional and reverse syphilis screening algorithms in a population with a low prevalence of syphilis. J Clin Microbiol 50:148–150. doi:10.1128/JCM.05636-11 22090407 PMC3256685

[10] French P, Gomberg M, Janier M, Schmidt B, van Voorst Vader P, Young H. 2009. IUSTI: 2008 European guidelines on the management of syphilis. Int J STD AIDS 20:300–309. doi:10.1258/ijsa.2008.008510 19386965

[11] Morshed MG, Singh AE. 2015. Recent trends in the serologic diagnosis of syphilis. Clin Vaccine Immunol 22:137–147. doi:10.1128/CVI.00681-14 25428245 PMC4308867

[12] Seed CR, Margaritis AR, Bolton WV, Kiely P, Parker S, Piscitelli L, Virology Subcommittee of the National Donor and Product Safety Committee, Australian Red Cross Blood Service. 2003. Improved efficiency of national HIV, HCV, and HTLV antibody testing algorithms based on sequential screening immunoassays. Transfusion 43:226–234. doi:10.1046/j.1537-2995.2003.00304.x 12559018

[13] Cheng A, Seed C, Das A, McCormack B, Gosbell I. 2023. P235 improved efficiency using sequential screening assays for Treponema allidum (syphilis) in blood donations. Vox Sang 118:119–379. doi:10.1111/vox.13433_1

[14] Conti G, Notari EP, Dodd RY, Kessler D, Custer B, Reik R, Lanteri MC, Hailu B, Yang H, Stramer SL, U.S. Transfusion-Transmissible Infections Monitoring System (TTIMS). 2024. Syphilis seroprevalence and incidence in US blood donors from 2020 to 2022. Transfusion 64:325–333. doi:10.1111/trf.17707 38180267 PMC10922865

[15] Tong M-L, Lin L-R, Liu L-L, Zhang H-L, Huang S-J, Chen Y-Y, Guo X-J, Xi Y, Liu L, Chen F-Y, Zhang Y-F, Zhang Q, Yang T-C. 2014. Analysis of 3 algorithms for syphilis serodiagnosis and implications for clinical management. Clin Infect Dis 58:1116–1124. doi:10.1093/cid/ciu087 24550376

